# Intermolecular Interactions in the Membrane Filtration of Highly Alkaline Steeping Lye

**DOI:** 10.3390/membranes11020088

**Published:** 2021-01-27

**Authors:** Klaus Schlackl, Richard Herchl, Lukas Almhofer, Robert H. Bischof, Karin Fackler, Wolfgang Samhaber

**Affiliations:** 1 Kompetenzzentrum Holz GmbH, 4040 Linz, Austria; l.almhofer@wood-kplus.at; 2 Lenzing AG, 4860 Lenzing, Austria; r.herchl@lenzing.com (R.H.); r.bischof@lenzing.com (R.H.B.); k.fackler@lenzing.com (K.F.); 3 Department of Process Engineering, Johannes Kepler University, 4040 Linz, Austria; wolfgang.samhaber@jku.at

**Keywords:** nanofiltration, ultrafiltration, steeping lye, hemicellulose, hydrogen bonds, intermolecular interactions

## Abstract

The reuse of steeping lye is crucial for the sustainable production of viscose fibers. Steeping lye contains hemicellulose and many alkaline degradation products, such as organic acids, so that its purification can be evaluated in terms of total organic carbon removal. When considering purification by membrane filtration, intermolecular interactions between hemicellulose and organic acids can strongly affect their retention efficiency. Herein, we give more insights into the ultrafiltration and nanofiltration of steeping lye and corresponding model solutions. Furthermore, we studied the impact of total organic carbon concentration, hemicellulose concentration and sodium hydroxide concentration on the membrane performance. Hydrogen bonds between hemicellulose and certain types of hydroxy acids increased the retention of the latter. In contrast, charge based repulsion forces led to a decreased retention of a certain type of hydroxy acids. It can be clearly shown that taking intermolecular interactions into account is highly important for the description of complex multicomponent mixtures. In addition, the results can be extended to other, highly alkaline process streams with organic content, such as Kraft pulping liquors.

## 1. Introduction

Cellulose is the most abundant biopolymer on earth. Wood is mainly composed of cellulose, hemicellulose, and lignin. Cellulose must be separated from hemicellulose and lignin during the production of dissolving pulp. Subsequently, the pulp is subjected to washing, bleaching and further treatment for use as dissolving grade pulp. In the production of viscose fibers, which utilizes dissolving wood pulp as raw material, several processing steps are conducted in highly concentrated sodium hydroxide solutions. In the steeping process, sodium hydroxide concentrations ranging from 17.5 to 19.5 w% are typically used for swelling as well as adjusting the molecular weight distribution of cellulose as a prerequisite for its xanthogenation [1]. In this stage, cellulose remains undissolved, while hemicellulose and further wood degradation products enter the solution. To ensure process efficiency, sodium hydroxide must be purified from hemicellulose and other wood degradation products and recycled for the steeping process. To further increase the economic and ecological benefits, hemicellulose must be converted to high value-added products. Dependent on the final product, various hemicellulose fractions and purities are required and thus different processes, such as ultrafiltration, nanofiltration or dialysis, can be favorable. For almost eight decades, dialysis processes were employed for the purification of sodium hydroxide [2,3]. However, dialysis technology is limited by large membrane areas and dilute process streams. Thus, the utilization of pressure-driven separation processes, such as ultrafiltration and nanofiltration, is preferred. However, a limited amount of commercially available membranes that can withstand harsh conditions, such as 19.5 w% sodium hydroxide, are available.

Basically, hemicellulose can either be converted to various products such as xylose, xylitol, and xylooligosaccharides (XOS) or it can be used as a carbon source for biofuel production [4]. Du [5] reported a novel application of hemicellulose for the formation of polyacrylamide–hemicellulose hybrid films. A number of studies reported the isolation of hemicellulose and its conversion to prebiotic XOS [6]. Griebl [7] investigated the isolation of hemicellulose from untreated steeping lye by precipitation in diluted sulfuric acid. Similar investigations were performed by Zhang [8] and Kabrelian [9]. Sihtola [10] reported a patent in which hemicellulose is precipitated using ethanol and subsequently filtered, followed by the evaporation of ethanol from the filtered steeping lye to obtain clean sodium hydroxide and ethanol; hence, both components can be reused in the process. To produce XOS, several combinations of membrane processes were published in patents. Deng published a process based on nanofiltration in dilution mode followed by electrodialysis [11] or diffusion dialysis [12] prior to the precipitation of hemicellulose with acids, with the subsequent enzymatic conversion of hemicellulose into XOS and final purification by filtration in dilution mode using ceramic membranes.

As the modification of these processes, instead of the dialysis process, several nanofiltration cycles can be performed [13,14].

Schlesinger examined different nanofiltration membranes and their suitability for the purification of steeping lye in terms of the total organic carbon (TOC) removal [15]. Singh reported the performance of ceramic ultrafiltration membranes [16] and polymeric ultrafiltration membranes [17] in terms of hemicellulose retention and long-term performance. In both cases, a membrane with a nominal molecular weight cut-off MWCO of 3 kDa exhibits a hemicellulose retention of about 0.68. All these investigations are performed with solutions derived from industrial processes. Prior to this study, the performance of the NP030 (Microdyn Nadir) membrane was investigated using synthetic solutions in terms of the sodium hydroxide–organic interactions [18].

Membrane performance is a key factor for several utilization processes of hemicellulose, as well as for the purification of steeping lye; however, several impact factors have not been investigated. Therefore, in this study, different liquors derived from an industrial steeping process are utilized, with focus on impacts of the β (hemicelluloses) to γ (hydroxy acids and sugar oligomers) ratio and sodium hydroxide concentration on the retention of polymeric ultrafiltration and nanofiltration membranes. The membranes used are categorized in ultrafiltration and nanofiltration membranes based on the manufacture’s specification. A clear differentiation cannot be conducted only based on the membrane’s MWCO, and the process solutions also need to be considered.

The focus was on detailed analysis of organic components, which allowed the first ever detailed investigation of intermolecular interactions between hemicelluloses and hydroxy acids in steeping lye. For this, we provide detailed analyses of the retention of sugars and several hydroxy acids, such as glucoisosaccharinic acid, xylo isosaccharinic acid, glycolic acid, etc., i.e., organic acids formed through alkaline polysaccharide degradation, which are leading constituents of the γ-cellulose fraction. Mozdyniewicz [19] extensively investigated the origin and formation of these substances during the steeping process as well as their chemical properties. Long-term membrane performance and specific fouling experiments were not conducted because these aspects were previously published by Schlesinger [16].

## 2. Materials and Methods

### 2.1. Material

#### 2.1.1. Synthetic Solutions

Synthetic solutions were prepared using succinic acid (>99.0% purity) and sodium hydroxide pellets with a >99.0% purity (VWR-International, Vienna, Austria).

#### 2.1.2. Industrial Process Streams

**Figure 1:**
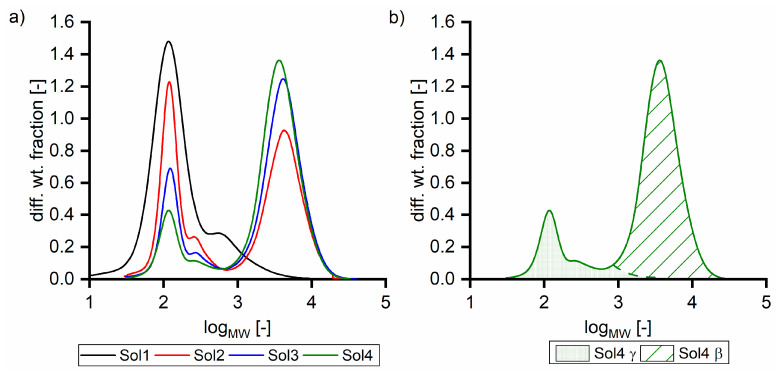
Size exclusion chromatograms (**a**) of different solutions and (**b**) of Sol4 separated in β and γ fractions.

In addition to synthetic solutions, solutions from an industrial viscose fiber process were used. Table 1 summarizes the compositions of five solutions (Sol1–5) with different TOC concentrations and varying β to γ ratios (see Section 2.2.3). Furthermore, dilutions of Sol5 with water and 5 mol L^−1^ sodium hydroxide were used. Depending on the sugar concentration, the recovery rate of analysis in relation to the TOC was between 75 and 95%, indicating that 75 to 95% of the total amount of carbon can be assigned to the analyzed components. The gap can be explained by measurement inaccuracy and the fact that some organic acid concentrations were under the detection limit. Furthermore, some organic acids were analyzed in a semi-quantitative way. Figure 1a shows the corresponding size exclusion chromatograms, and Figure 1b shows the separation of Sol4 in β and γ fractions. Sol4 was prepared from Sol5 by diluting with 5 mol L^−1^ sodium hydroxide. Thus, Figure 1a does not show Sol5. The sizes of the two peaks of the bimodal distribution did not adequately represent the concentration ratios, as hydroxy acids, sugars, and hemicellulose exhibit noticeable differences in the detector response. Nevertheless, differences in the molecular mass distribution were observed for the used solutions.

#### 2.1.3. Membranes

Various commercially available and alkaline robust polymeric ultrafiltration and nanofiltration membranes purchased from Microdyn Nadir (Ludwigsburg, Germany) were used for the experiments. In addition, a highly alkaline stable nanofiltration membrane (NF090801) from SolSep BV (Apeldoorn, The Netherlands) was used. Table 2 summarizes the relevant membrane properties as specified by the manufacturer. The ultrafiltration membranes (UP and UH) were chosen, as they are all polyethersulfone (PES)—based with similar basic structure. The membranes therefore have the same chemical properties and differ only in their MWCO. The NP010 membrane was considered in this study as if it were an ultrafiltration membrane, although its MWCO is in the nanofiltration range. However, due to the goal of this study it is more suitable to compare it with the ultrafiltration membranes than with the nanofiltration membranes.

### 2.2. Method

#### 2.2.1. Membrane Filtration

Laboratory-scale membrane filtration experiments were performed using an OSMO Memcell 3 flat-sheet filtration device; a detailed description of the device was published previously [19]. Filtration was performed in the tangential flow mode with three filtration cells arranged in parallel. The active filtration area was 80 cm^2^ per cell. The tangential flow velocity and temperature were set to 1.0 m s^−1^ and 40 °C, respectively. To maintain a constant feed concentration, experiments were performed in the total recirculation mode. The sample volume was sufficiently low to ensure negligible changes in the feed concentration.

The performance of membranes from different sheets varied slightly; thus, experiments are clustered into groups in which the same membranes are used. For pretreatment, membranes were rinsed with deionized water for 72 h. The maximum pressure during the pretreatment was equal to the highest pressure in consecutive experiments, thus hypothetical membrane pre–compaction occurred during pretreatment. Sulfate retention (0.1 mol L^−1^ sodium sulfate, 40 °C, 25 bar) experiments were performed with the nanofiltration membranes before and after a set of experiments to monitor changes in the membrane behavior. Accepted values for sulfate retention were 0.80 ± 0.05 for the NP030 membrane and 0.90 ± 0.05 for the NF090801 membrane. All new membranes fulfilled these requirements. If the sulfate retention of a spent membrane was out of its range after a set of experiments, the membrane was substituted by a new one and the set of experiments was repeated with the new membrane.

#### 2.2.2. Analytical Methods

##### Total Organic Carbon (β)

TOC measurements were performed according the Austrian Standard ÖNORM EN 1484 (2019).

##### Chromatography

The quantification of sugars was performed on chromatography, as reported previously [20]. In this study, the sample preparation additionally includes total hydrolysis; thus, the sugars can be quantified as monomers. Results of monosaccharide measurements were corrected for hydrolysis water, when concentrations of polymeric hemicelluloses were provided.

Succinic acid was quantified by high-performance liquid chromatography (HPLC) analysis, as reported previously [18].

Derivatization of hydroxy acids was performed as follows: first, 200 µL of the sample was subjected to lyophilization at −54 °C for 48 h. Subsequently, 900 µL of anhydrous pyridine was added to the sample, and the sample was heated to 70 °C for 1 h. Next, the sample was cooled to room temperature, and 100 µL of N,O-bis(trimethylsilyl)trifluoroacetamide (BSTFA) was added as the derivatization agent. During derivatization, the temperature was maintained at 70 °C for 1 h; finally, the sample was cooled to room temperature. Analyses were performed on a Shimadzu QP2010 gas chromatography system (Shimadzu Europa, Duisburg, Germany), which comprised an HP5-MS column (60 m length × 0.25 mm inner diameter × 0.25 µm film thickness; J&W Scientific, Folsom, CA, USA) coupled with a Shimadzu QP2020 dual stage mass spectrometer. The following operation parameters were utilized during analyses: mode: split injection; constant column flow: 1.2 mL min^−1^; carrier gas: helium; purge flow: 3.0 mL min^−1^; split ratio: 1:10; total flow: 15.2 mL min^−1^; injector temperature: 250 °C; and temperature gradient profile: 50 °C (15 min), then 5 °C min^−1^ to 300 °C (5 min).

The mass detector was operated in the electron ionization (EI)-mode (ionization energy of 70 eV at 1.13 × 10^−7^ Pa) with an ion source temperature of 200 °C and a transfer line temperature of 250 °C. Data were acquired in the scan mode, ranging from 45 to 500 *m*/*z*. Aliquots of 1 µL were injected by an AOC 6000 autosampler (Shimadzu Europa, Duisburg, Germany). The National Institute of Standards and Technology (NIST) database was utilized for compound identification. Evaluation was performed in a semi-quantitative manner by using standards for lactic acid, glycolic acid, and glucoisosaccharinic acid. For the other components, evaluation was directly performed from peak areas by applying detector responses for substances with similar structures and known detector responses.

##### Size Exclusion Chromatography (SEC)

Size-exclusion chromatography (SEC) measurement was performed as described previously [21].

#### 2.2.3. β and γ Fractionation

In the wood-based fiber industry, cellulose is typically classified as α, β and γ forms. The α fraction represents cellulose that is insoluble in 18 w% caustic solutions. The β fraction is defined as soluble in 18 w% sodium hydroxide but insoluble under acidic conditions. Furthermore, the β fraction mainly consists of hemicellulose and degraded cellulose; thus, it comprises pentose and hexose oligomers. On the other hand, the γ fraction is the fraction that remains soluble after acidification. It contains short sugar oligomers and further wood degradation products such as various hydroxy acids [19].

The β and γ fractionation was performed as follows: first, samples were diluted with deionized water in a ratio of 1:1. Subsequently, 40 mL of sulfuric acid (2.375 mol L^−1^) was added in a 100 mL volumetric flask. Next, 50 mL of the diluted sample was added into the flask, and precipitation was performed for 5 min while stirring. The flask was filled to 100 mL with sulfuric acid and stirred for another 25 min. Finally, the samples were filtered using a 0.45 µm polytetrafluoroethylene (PTFE) filter.

TOC measurements were performed using the diluted sample and filtrate. Thus, the amount of γ and β fractions can be calculated as shown in Equations (1) and (2), respectively.

(1) $$\gamma =\frac{2{\mathrm{TOC}}_{\mathrm{filtrate}}}{{\mathrm{TOC}}_{\mathrm{DS}}}\;\times \;100\;\left[\%\right]$$

(2) β=100−γ

TOC_filtrate_ and TOC_DS_ represent the TOC concentrations of the filtrate and of the diluted sample, respectively, in units of g L^−1^.

## 3. Results

The results section is divided into three parts. In the first part, the intermolecular interactions that play an important role in the processing of steeping lye are discussed. In the second part, the performance of ultrafiltration membranes for hemicellulose and sugar recovery from steeping lye plays the main role, and in the last part, the application of nanofiltration membranes for the purification of steeping lye is revealed.

### 3.1. Intermolecular Interactions

Two counteracting intermolecular interactions need to be taken into account to explain the unexpected retention behavior of steeping lye.

#### 3.1.1. Hydrogen Bond Formation

**Figure 2:**
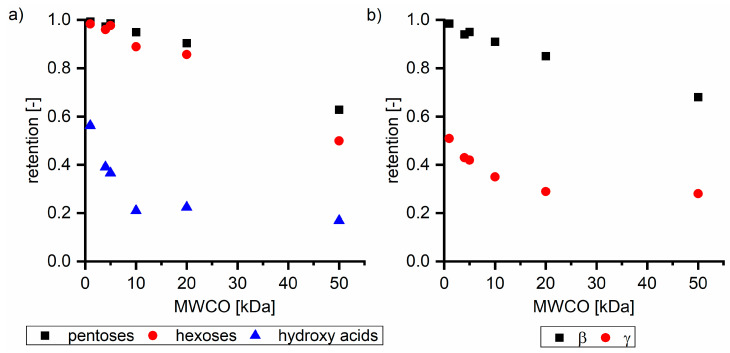
Retention of (**a**) pentoses, hexoses, and hydroxy acids and (**b**) β and γ fractions dependent on the MWCO of ultrafiltration membranes, exemplified with Sol2. NP010, UH004, and UP005 membranes were used at a pressure of 15 bar; UP010 and UP020 at a pressure of 5 bar; and UH005 at a pressure of 3 bar.

Figure 2a shows the retention of sugar oligomers analyzed as pentoses and hexoses, and, additionally, hydroxy acids with different ultrafiltration membranes based on their MWCO examined with Sol2 as the feed solution. The retention of sugars was almost 1.0 with the NP010 membrane, but it decreased to 0.50 (hexoses) and 0.63 (pentoses) with the UH050 membrane. In all the performed experiments, the retention of pentoses was higher compared to hexoses retention. Both types of sugars were present in the form of oligomers or polymers. Differences in retention can be explained either by different degrees of polymerization or by charge effects. Hexoses are mainly derived from the degradation of cellulose; thus, molecules do not contain charged groups. Pentoses are mainly hemicellulose degradation products and are known to contain a high number of negatively charged carboxyl groups (in the form of 4-methyl glucuronic acid substitutions). Figure 2b shows the dependence of the retention of the β and γ fractions on the membrane’s MWCO. As expected based on molecular weights, the retention of the β fraction was more than twice as high as the retention of the γ fraction. With the increase in MWCO, the retention of both fractions decreased. Although the molecular weights of the γ components (<180 g mol^−1^) were significantly below the MWCO of some membranes, the γ components exhibited a retention of 0.28 with the 50 kDa membrane, which can be explained by intermolecular interactions between β and γ fractions. A β free solution did not show any significant retention on membranes with a MWCO higher than 10 kDa as shown in Section 3.2.2. Thus, charge based interactions between the negatively charged membrane and the hydroxy acids cannot be responsible for the unexpected high retention of γ components on ultrafiltration membranes.

Table 3 summarizes the concentrations of hydroxy acids in Sol2 and their retention on the tested membrane types. The pK_A_ values of the present hydroxy acids are in between 3.5 and 5.1, thus all the acids can be considered as completely dissociated in strong alkaline conditions. As already shown in Figure 2a, with increasing MWCO, the overall hydroxy acid retention decreased. However, the retention behavior of the individual acids showed extreme variation. Hydrogen bonds between the hemicelluloses and hydroxy acids appear to affect the retention of the latter. Similar observations were previously published for the nanofiltration of wood hydrolysates, in which hydrogen bonds between lignosulfonate and furan derivatives increased the retention of some furan derivatives, dependent on the oxygen configuration in the molecule [21]. The capability to form hydrogen bonds with different biopolymers is essential for the natural function of xylan, and this was already studied by several authors with different prospects. Pereira [22] gives an overview of influencing factors. The number of hydroxyl groups per molecule and their steric configuration considerably affect the retention behavior. Short-chain organic molecules such as lactic acid, glycolic acid, or glycerol comprise a comparably high number of hydroxyl groups in relation to their molecular mass. Furthermore, these molecules can orientate their hydroxyl groups in a manner to facilitate hydrogen-bond formation with the hemicelluloses.

**Figure 3:**
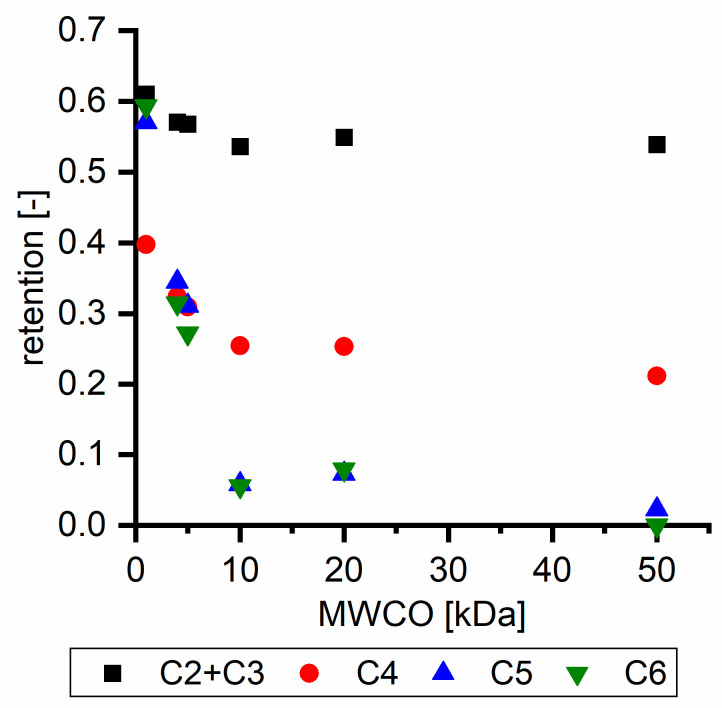
Retention of groups of hydroxy acids clustered based on their chain length.

Hydroxy acids can be grouped based on their number of carbon atoms per molecule. The retention of the individual clusters shows that larger molecules did not consistently show higher retention with all ultrafiltration membranes (Figure 3). To describe this behavior, two aspects need to be considered: the retention capability of the membrane for each molecule and the interaction capability of molecules with hemicelluloses. Tight membranes exhibited noticeable retention for pure C5 and C6 hydroxy acids (data not shown); thus, these components exhibit high retention in the mixture as well. Membranes with a high MWCO exhibited a lower retention capability for pure substances, and owing to the absence of noticeable interactions with hemicelluloses, the retention decreased in the mixture as well. Compared with that of C5 and C6, the retention of pure C2 and C3 hydroxy acids was considerably lower for NP010. However, owing to their interactions with highly retained hemicelluloses, the retention in the mixture was in the same range as that observed for C5 and C6 acids. Furthermore, the retention of C2 and C3 was less dependent on the MWCO of the membranes. Hydroxy acids with four carbon atoms exhibited a cumulative retention behavior between short-chain (C2 and C3) and long-chain (C5 and C6) hydroxy acids.

Nevertheless, the retention of individual C4 acids considerably varied with the increase in the number of isomers. Hence, there are molecules with higher and lower tendencies to form hydrogen bonds with hemicelluloses. With the further increase in the chain length, steric hindrance may play a more pronounced role; thus, the capability of hydrogen-bond formation decreases, leading to the decrease in the observed retention.

#### 3.1.2. Charge based Interactions

**Figure 4:**
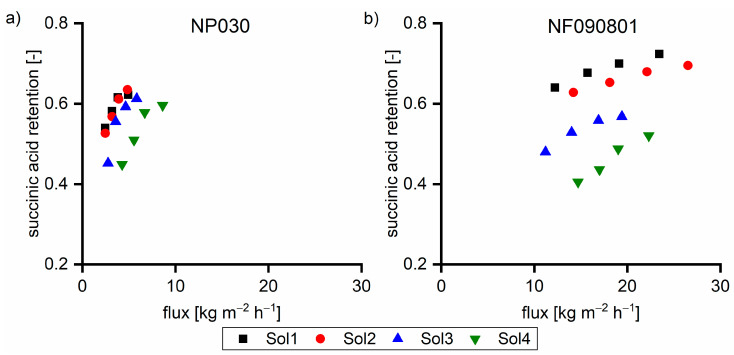
Succinic acid retention of 5 g L^−1^ succinic acid in various feed solutions at 40 °C with (**a**) NP030 and (**b**) NF090801. The applied pressure values were 35, 30, 25, and 20 bar, respectively.

Hemicellulose comprised ~0.3 mmol g^−1^ of carboxylic acid groups, which underwent dissociation due to the high pH value. The number of hydroxy groups linked to the hemicellulose exhibited the same order of magnitude as the overall negative charge from hydroxy acids. Due to the extremely high retention, hemicelluloses led to a proportional higher anion concentration on the feed side. Due to the Donnan potential, permeable anions such as hydroxy acid anions are forced to move to the permeate side to equalize the charge balance. The coupling of cation and anion retention in highly caustic solutions was previously published using succinic acid as the model substance [19]. To prove the hypothesis of the effect of hemicellulose on the hydroxy acid retention, solutions (Sol1–Sol4) were spiked with 5 g L^−1^ succinic acid, an organic acid with no hydroxyl group, to prevent hydrogen bonding. Figure 4 shows the retention of succinic acid as a function of the hemicellulose concentration. The increase in the hemicellulose concentration led to the decrease in the succinic acid retention, which supports our hypothesis.

### 3.2. Performance of Ultrafiltration Membranes

#### 3.2.1. Flux Behavior of Ultrafiltration Membranes

**Figure 5:**
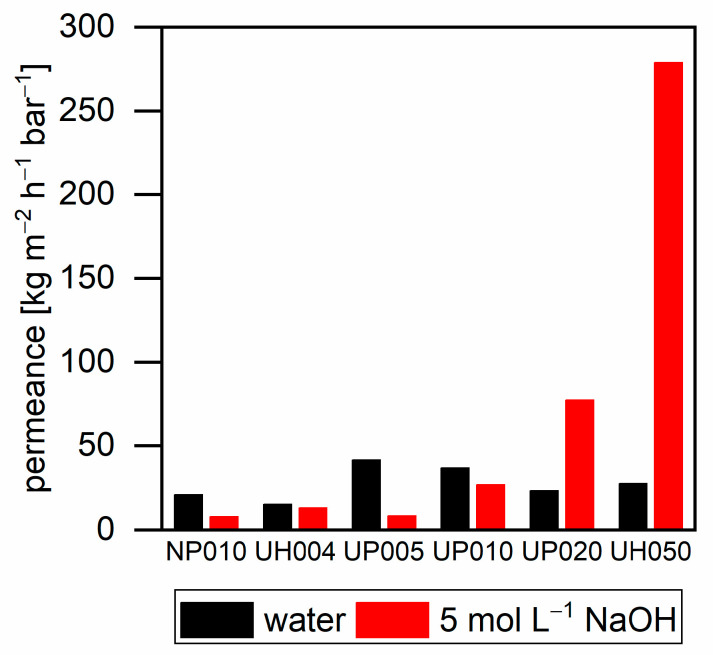
Permeance of water and 5 mol L^−1^ sodium hydroxide with different ultrafiltration membranes at 40 °C. NP010 and UH004 at a pressure of 15 bar; UP005, UP010, and UP020 at a pressure of 10 bar; and UH050 at a pressure of 5 bar.

Figure 5 shows the pure water flux and the flux of 5 mol L^−1^ sodium hydroxide through the used ultrafiltration membranes. Ultrafiltration membranes with a low MWCO exhibited higher values for pure water flux compared to sodium hydroxide. Their lower flux can be explained by the higher viscosity of sodium hydroxide solutions. By the increase in the MWCO of the membranes, the sodium hydroxide flux increased in comparison to the pure water flux; thus, the UH050 membrane exhibits a 10-fold higher sodium hydroxide flux than that of pure water. The higher flux of the more viscous sodium hydroxide can be explained by caustic membrane swelling. Different research groups investigated this phenomenon of polymeric membranes [23,24,25]. Swelling may occur with the low MWCO membranes as well; however, for these membranes, the higher viscosity of sodium hydroxide exhibited a higher impact on the flux than membrane swelling.

#### 3.2.2. Retention of Ultrafiltration Membranes

**Figure 6:**
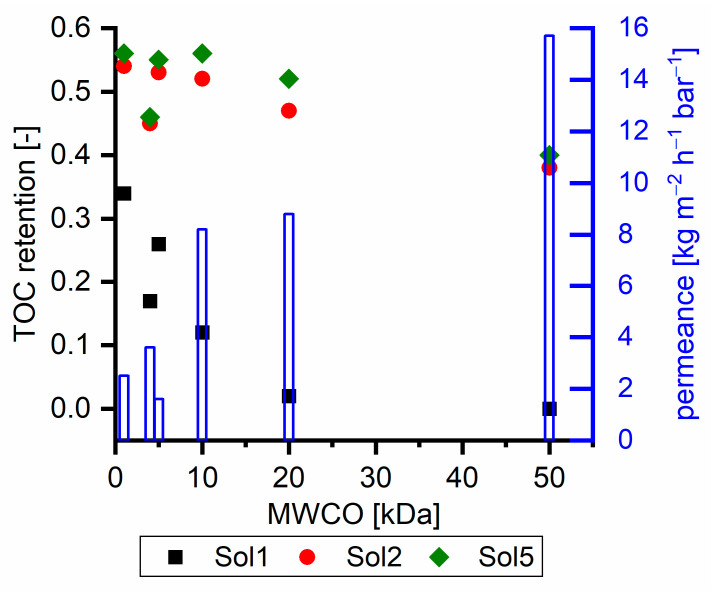
TOC retention of different ultrafiltration membranes with dependency on their MWCO examined with different solutions. The blue bars show the corresponding permeance examined with Sol1. NP010, UH004, and UP005 at a pressure of 15 bar; UP010 and UP020 at a pressure of 5 bar; and UH005 at a pressure of 3 bar.

Figure 6 shows the TOC retention of ultrafiltration membranes with different feed solutions at 40 °C. Retention was examined at different pressures to work within the respective nominal pressure range for each membrane. All membranes were tested at different pressures and the membranes showed expected relations between pressure, flux and retention. Thus, the shown data sets are representative to compare the different membranes without significant pre-compaction or any further modification of the membranes. The retention of Sol1 decreased from 0.34 to 0, which indicates that pure γ components are not retained by a 50 kDa MWCO membrane. Sol2 and Sol5 exhibited higher retention with tighter membranes, and their retention was still about 0.40 with a 50 kDa membrane. Related to the SEC measurements (Figure 1), this retention seems to be too high, as, e.g., for Sol2 75% of TOC has a molecular weight less than 1000 g mol^−1^ In other words, the γ retention with Sol2 is 0.28 (Figure 2) compared to 0 with Sol1. The reason for this behavior is the intermolecular interactions, which are discussed in Section 3.1. Additionally, the absence of retention of Sol1 with the 50 kDa membrane indicates that membrane γ component interactions can be neglected with the chosen setup.

Furthermore, the retention of the UH004 membrane, a hydrophilized PES membrane, was less than that of the UP005 membrane (PES) for all of the three tested solutions. However, the flux of the UH004 membrane was more than twice as high as that of the UP005 membrane with Sol1 and Sol2 and ~70% higher with Sol5 (data not shown).

#### 3.2.3. Criteria for Ultrafiltration Membrane Selection

For the separation and purification of hemicelluloses from steeping lye, the β and γ interactions need to be considered. Depending on the subsequent utilization of hemicellulose, two of the tested membranes were favorable. The UP005 membrane exhibited the highest potential for the separation of sugars from hydroxy acids and sodium hydroxide (Figure 2a). The retention of pentose was 0.95, with a concurrent hydroxy acid retention of 0.21. The NP010 membrane was favorable for the separation of a pure hemicellulose fraction with a certain minimum molecular weight (β fraction) (Figure 2b). A β retention of 0.98 with a corresponding γ retention of 0.51 permitted the purification of the β fraction, without the extensive loss of β components, applying ultrafiltration in dilution mode.

### 3.3. Performance Study of Nanofiltration Membranes

#### 3.3.1. Flux Behavior of Nanofiltration Membranes

**Figure 7:**
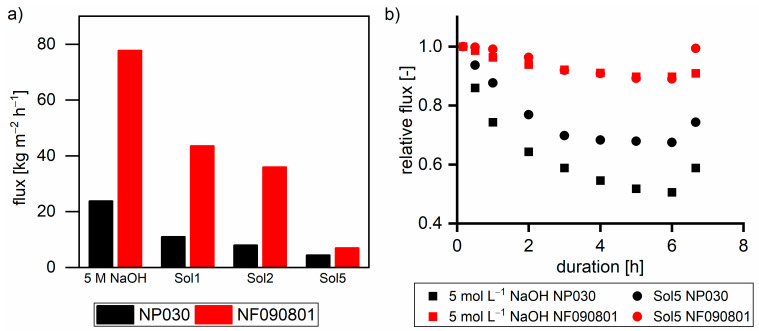
(**a**) Flux of nanofiltration membranes with different solutions at 30 bar and 40 °C. (**b**) Relative flux related to the initial flux with two solutions. Last data points show the relative flux after pressureless membrane rinsing for 30 min.

Figure 7a shows the flux of nanofiltration membranes with different solutions at 40 °C and 30 bar. As can be observed, the flux decreased with the increase in the TOC and β concentration. Furthermore, the flux was always greater with NF090801 than that with NP030. The difference in the flux of both membranes decreased with the increase in the TOC concentration of the feed solutions.

Figure 7b shows the relative decline in the flux over time with respect to the flux after 10 min (t = 0) with different solutions. For NP030 with 5 mol L^−1^ sodium hydroxide, the relative flux decline was higher. Hence, compared to NF090801, NP030 exhibits a higher tendency for membrane compaction, which is also stated in the material data sheet of NP030. Moreover, higher TOC and β concentrations led to a lower relative flux decrease with both membranes due to the reversible formation of a secondary filtration layer built up from hemicelluloses. The layer immediately lowered the flux; thus, the impact of membrane compaction is less pronounced. The deconstruction of the hemicellulose layer during 30 min of pressure-less membrane rinsing led to a flux recovery of 74% and 99% with respect to t = 0 for NP030 and NF090801, respectively. A detailed study on the fouling behavior of steeping lye including the NP030 membrane was provided by Schlesinger [26].

#### 3.3.2. Retention Behavior of Nanofiltration Membranes

**Figure 8:**
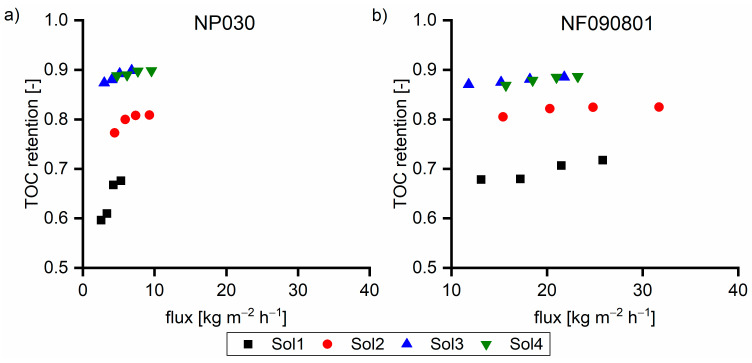
TOC retention of (**a**) NP030 and (**b**) NF090801 with various feed solutions at 40 °C. The applied pressure values were 35, 30, 25, and 20 bar, respectively.

Figure 8 shows the TOC retention performance of the two membranes. In all cases, the flux with the NF090801 membrane was greater than that with the NP030 membrane. In solutions without the β fraction (Sol1), the retention of the NF090801 membrane was between 0.69 and 0.72. For the NP030 membrane, the retention was between 0.60 and 0.68. Furthermore, the flux dependence of the retention of the NP030 membrane was considerably greater than that of the NF090801 membrane. With Sol2, both membranes exhibited higher retention, and the performance differences between the two membranes decreased. Moreover, the flux dependence on retention was less for the NP030 membrane. The membranes exhibited similar retention behavior at elevated β concentrations (Sol3 and Sol4). In conclusion, the membranes varied in terms of their retention of small γ components. With Sol3 and Sol4 with 39% and 45% of the β fraction, respectively, the membranes exhibited almost identical behavior.

**Figure 9:**
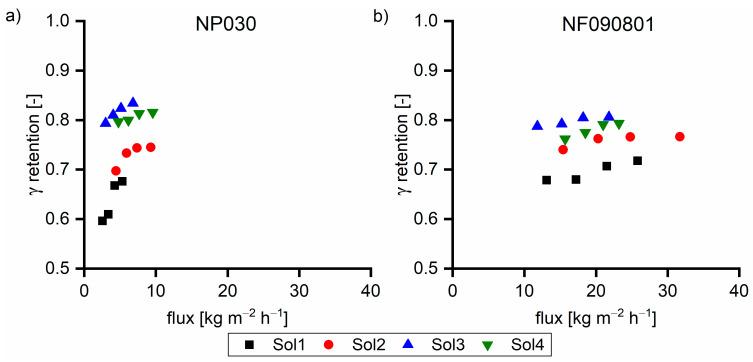
γ retention of (**a**) NP030 and (**b**) NF090801 with various feed solutions at 40 °C. The applied pressure values were 35, 30, 25, and 20 bar, respectively.

Taking a closer look at the retention shown in Figure 9, the γ retention initially increased with the increase in the β concentration (Sol1–Sol3). The further increase in the β concentration led to the decrease in the γ retention as the TOC retention remained constant (Figure 8). In all cases, the sugar retention was greater than 0.99 (data not shown). Hence, the retention of hydroxy acids must have changed. With increased β concentration, the charge-based repulsion forces became more pronounced in relation to the hydrogen bond-based adhesive forces.

Table 4 summarizes the retention of hydroxy acids based on their chain length with different feed solutions. With the increase in the β concentration, the retention of C2 and C3 hydroxy acids increased, whereas the retention of the C4–C6 hydroxy acids decreased. The increase or decrease in the overall hydroxy acid retention in the presence of hemicellulose depends on the concentration and on the hydroxy acid species. Hence, the hydroxy acid retention increases for acids with a high capability of hydrogen bond formation in the presence of hemicelluloses, while the overall hydroxy acid retention decreases for acids with a low hydrogen bond formation capability.

#### 3.3.3. The Impact of Dilution

The influence of the sodium hydroxide concentration on the membrane behavior was tested with Sol5. For this purpose, the solution was stepwise diluted with deionized water or 5 mol L^−1^ sodium hydroxide. The dilution from 5 to 2 mol L^−1^ sodium hydroxide led to a flux increase at 30 bar from 4.5 to 19.6 and from 8.8 to 52.0 kg m^−2^ h^−1^ for NP030 and NF090801, respectively. The higher the dilution rate, the more pronounced the flux increase. Hence, the dilution from 5 to 3 mol L^−1^ sodium hydroxide exhibits less impact on the flux than the dilution from 3 to 2 mol L^−1^ sodium hydroxide (Table 5).

**Figure 10:**
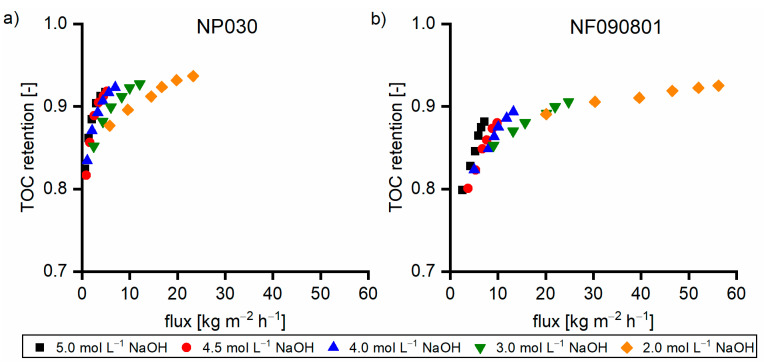
TOC retention of Sol5 with various dilution rates with water for (**a**) NP030 and (**b**) NF090801.

Figure 10a,b show the TOC retention for the NP030 and NF090801 membranes, respectively. With the decrease in the sodium hydroxide concentration, retention increased due to the increase in flux. The comparison at the same flux revealed that the retention is high with a high concentration of TOC in the feed solution. This behavior was observed for both membranes. The higher the dilution rate, the lower the pressure required to facilitate a certain flux. At low pressures, the membrane compaction was not as pronounced; thus, the retention was slightly low. Identical behavior was observed for the UP020 membrane in a pressure range from 1 to 10 bar (data not shown).

Diluting Sol5 with 5 mol L^−1^ sodium hydroxide instead of water led to a lower increase in flux (Table 5). Furthermore, the retention did not exhibit a noticeable dependency on the TOC concentration in the range from 60 to 8 g L^−1^ (data not shown).

## 4. Conclusions

Sodium hydroxide flux is far more dependent on MWCO than pure water flux. Consequently, depending on the used membrane, an optimized sodium hydroxide concentration can be found for the treatment of diluted steeping lye to enhance membrane performance. For the purification of steeping lye, in terms of TOC removal, the NF090801 membrane is favorable because of its higher flux by comparable retention to the NP030 membrane. All ultrafiltration membranes exhibit insufficient TOC retention.

For the separation and purification of sugars, the UP005 membrane is the most favorable as it exhibits the highest selectivity in terms of sugar (0.95) and hydroxy acid (0.21) retention. For the recovery of hemicelluloses from steeping lye, a diluted ultrafiltration process with the NP010 membrane is most favorable as the β retention is 0.98 and the γ retention is 0.51.

Moreover, a closer look at the retention data leads to the conclusion that hemicellulose–hydroxy acid interactions have a higher impact on the hydroxy acid retention than the actual retention at the active membrane layer. This underlines the importance of investigations on intermolecular interactions in nano and ultrafiltration processes.

The increase or decrease in hydroxy acid retention in the presence of hemicelluloses strongly depends on the acid type and structure. It seems that the number of hydroxyl groups per acid molecule and steric effects are the major influencing factors. It can be concluded that the insights of the present study support the design and improvement of TOC removal and hemicellulose purification processes based on highly alkaline process liquors. Furthermore, it can be pointed out that intermolecular interactions should be generally considered to be more important in studies dealing with organic multicomponent solutions.

## Figures and Tables

**Table 1:** Composition of the used five streams.

Parameter	Sol1	Sol2	Sol3	Sol4	Sol5
c_NaOH_ [mol L^−1^]	5	5	5	5	5
TOC [g L^−1^]	8	16	30	30	60
β [%]	0	25	39	45	45
γ [%]	100	75	61	55	55
hexoses [g L^−1^]	0	0.5	1.8	2.1	4.1
pentoses [g L^−1^]	0	8.2	27.6	31.8	63.5
hydroxy acids * [g L^−1^]	17.3	22.0	24.8	15.5	30.1

* “hydroxy acids” include various hydroxy acids and glycerol, which was analyzed along with hydroxy acids.

**Table 2:** Membrane properties given by the manufacturer.

Property	NP030	NP010	UH004	UP005	UP010	UP020	UH050	NF09081
Support material	PES	PES	PESH	PES	PES	PES	PESH	PET
Surface material	PES	PES	PESH	PES	PES	PES	PESH	SPEEK *
Maximum temperature [°C]	95	95	95	95	95	95	95	80
Recommended pH range	0–14	0–14	0–14	0–14	0–14	0–14	0–14	up to 14
MWCO [g mol^−1^]	30 **	10 ***	4000	5000	10,000	20,000	50,000	50 **
Water permeance [L m^−2^ h^−1^ bar^−1^]	1	5	6.8	10.2	50.3	70.3	85	1–2

* The manufacturer did not provide any information about the polymer in the activelayer. In-house infrared spectroscopy measurements showed agreement with polyether ether ketone (PEEK) and PES; thus, sulfonated poly (ether ether ketone) (SPEEK) might be the membrane polymer. ** NaCl retention in %; *** NaCl retention in %; the corresponding MWCO is assumed with 1000 Da; PET stands for polyethylene terephthalate.

**Table 3:** Feed concentration and retention of detected hydroxy acids with solution Sol2. Negative retention values may be attributed to measurement accuracy. The used pressures were 10 bar for NP010, UH004, and UP005; 5 bar for UP010 and UP020; and 3 bar for UH050. The colors mark components with high retention (green) and low retention (yellow).

	MW [Da]		c_Feed_ [mg_TOC_ L^−1^]	Retention [−]					
				NP010	UH004	UP005	UP010	UP020	UH050
glycolic acid	76.1	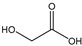	469	0.54	0.60	0.64	0.51	0.52	0.51
lactic acid	90.1	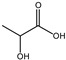	1324	0.60	0.52	0.51	0.52	0.54	0.51
3-hydroxypropanoic acid	90.1	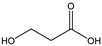	197	0.64	0.63	0.57	0.47	0.48	0.46
glycerol	92.1	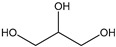	186	0.85	0.79	0.82	0.78	0.76	0.88
hydroxyisobutyric acid	104.1	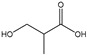	160	0.26	0.27	0.10	0.01	0.05	0.02
4-hydroxybutanoic acid	104.1	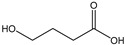	257	0.16	0.03	−0.01	0.00	0.04	0.00
2,3-dihydroxy-2-methylpropanoic acid	120.1	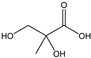	104	0.52	0.52	0.46	0.37	0.39	0.36
malic acid	135.1	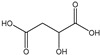	86	0.51	0.71	0.79	0.77	0.84	0.80
2,4-dihydroxybutanoic acid	120.1	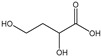	263	0.74	0.71	0.68	0.61	0.62	0.59
3,4-dihydroxybutanoic acid	120.1	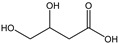	354	0.32	0.13	0.20	0.12	0.05	−0.01
4-hydroxy-pent-3-enoic acid	116.1	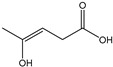	203	0.29	0.25	0.16	0.01	0.03	−0.01
2-hydroxypentanedionic acid	148.1	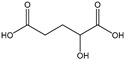	283	0.31	0.34	0.28	0.27	0.35	0.22
xyloisosaccharinic acid	150.1	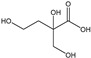	2030	0.64	0.36	0.33	0.02	0.03	−0.01
3-deoxy-xylaric acid	164.1	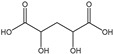	47	0.34	0.28	0.26	0.58	0.58	0.49
glucoisosaccharinic acid	180.2	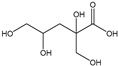	2431	0.59	0.31	0.27	0.06	0.08	−0.03
Hydroxy acid retention				0.56	0.39	0.37	0.21	0.22	0.17

**Table 4:** Dependence of the retention of hydroxy acids on their chain length as measured in various solutions.

	NP030			NF090801		
	Sol1	Sol2	Sol4	Sol1	Sol2	Sol4
C2+C3	0.54	0.75	0.72	0.61	0.77	0.72
C4	0.58	0.49	0.20	0.58	0.62	0.28
C5	0.73	0.75	0.55	0.77	0.69	0.45
C6	0.69	0.68	0.54	0.73	0.68	0.61

**Table 5:** Permeance of NP030 and NF090801 with Sol5 in various dilution rates.

NaOH [mol L^−1^]	TOC [g L^−1^]	Permeance [kg m^−2^ h^−1^ bar^−1^]	
		NP030	NF090801
5.0	60	0.13	0.23
4.5	54	0.14	0.30
4.0	48	0.18	0.41
3.0	36	0.33	0.76
2.0	24	0.67	1.76
5.0	30	0.16	0.66
5.0	16	0.24	1.08
5.0	8	0.27	1.18

## Data Availability

The data presented in this study are available on request from the corresponding author.
